# Governing Collaborative Healthcare Improvement: Lessons From an Atlantic Canadian Case

**DOI:** 10.15171/ijhpm.2017.60

**Published:** 2017-05-16

**Authors:** Meghan Rossiter, Jennifer Verma, Jean-Louis Denis, Stephen Samis, Richard Wedge, Chris Power

**Affiliations:** ^1^ Canadian Foundation for Healthcare Improvement, Ottawa, ON, Canada.; ^2^ Canada Research Chair on Governance and Transformation in Health Care Organizations and Systems, Université de Montréal-CRCHUM, Montreal, QC, Canada.; ^3^ Health Prince Edward Island, Charlottetown, PE, Canada.; ^4^ Canadian Patient Safety Institute, Ottawa, ON, Canada.

**Keywords:** Governance, Executive Leadership, Systems Change, Quality Improvement, Chronic Disease, Healthcare Improvement

## Abstract

The Atlantic Healthcare Collaboration for Innovation and Improvement in Chronic Disease (AHC) Quality Improvement Collaborative (QIC) in Eastern Canada provided an approach to spur system-level reform across multiple health systems for patients and families living with chronic disease. Developed and led by senior executives with a unique governance approach and involving clinical front-line teams, the AHC serves as a practical example of leadership creating and driving momentum for achieving success in collaborative health system improvements.

## Introduction

Quality improvement collaboratives (QICs) are becoming vital for disseminating improvements and bringing together healthcare organizations, frontline providers and patients toward common improvement objectives. While QICs provide structure and create shared improvement expectations,^1^ leadership and governance frameworks vary. To increase institutional support and sustainability, QICs require effective governance capacity.^2^ This article examines one approach to governing collaborative health system improvement with specific attention to:

- Development of a QIC *governance structure* through initiating a charter agreement, forming an executive committee among senior leadership of healthcare delivery organizations, and cost-sharing opportunities and strategies;
- Multi-dimensional (across systems) and -directional (top-down, bottom-up) *governance capacity* involving 17 regional health systems with shared responsibility, in partnership with a pan-Canadian improvement agency, endorsed by four provincial departments of health, with frontline clinicians and managers leading improvements; and
- Creating continued interest in *sustaining improvement* – be it the original interventions and/or capacity for quality improvement (QI), beyond the lifespan of the collaborative.

## Background

Canadian healthcare is a decentralized, complex, primarily publicly funded model dispersed across 10 provinces, three territories and the federal government.^3^ The Canadian Foundation for Healthcare Improvement (CFHI) is a non-profit, federally funded agency that accelerates spread of proven innovations across Canada by supporting healthcare organizations to adapt, implement and measure improvements in patient care, population health and value-for-money.

Facing escalating rates of chronic disease and rising healthcare costs, health systems across the four Atlantic provinces forged a QIC with CFHI to build capacity for more patient- and family-centred and sustainable care. The Atlantic Healthcare Collaboration for Innovation and Improvement in Chronic Disease (AHC) involved care teams working to design, implement and evaluate chronic care improvements (focusing on diabetes, mental health, chronic obstructive pulmonary disease and multimorbidity) and carrying out “disruptive changes” that challenged usual approaches to care.^3^

## I. Governance Structure

The AHC was designed and governed collectively by CFHI with chief executives and senior leaders from five participating Atlantic healthcare delivery organizations – forging the AHC Executive Committee (AHCEC). The AHCEC brought together key regional personnel to set a shared vision for achieving QI in chronic care, outlined in a charter agreement^4^ and signed by all 17 healthcare delivery regional health authority (RHA) CEOs and CFHI. The AHCEC members co-sponsored the QIC and were accountable for enacting the charter on behalf of all 18 signatories as per the agreed upon Terms of Reference.^5^ Core responsibilities included:

- Setting the direction for the QIC by selecting priority areas;
- Liaising with fellow RHA CEOs and leaders within their province to advise on activities; raising concerns or suggestions;
- Advising on policy changes for system-level improvements, allocating QIC resources, and strategies to enable success; and
- Acting as a forum for sharing information, assessing needs, responding to outcomes, evaluating success and adopting and spreading effective innovations.

A charter holds organizations and their leadership accountable for their QI efforts; collaboration toward common improvement goals is encouraged.^6^ Table 1 compares the features of an effective charter, defined by a Canadian CEO forum attended by more than 120 CEOs, policy-makers and experts,^5^ with the AHC charter^5^ and CFHI’s six levers for healthcare improvement framework, which informed the basis of the structure taken to the AHC.^7^ These features align with the six domains for leading improvement outlined in the Institute for Healthcare Improvement’s (IHI’s) High-Impact Leadership Framework, which outlines effective strategies for governing QI at an executive-level^8^:

**Table 1:** Comparison of Features of the AHC Charter and Elements of an Effective Charter

**Features of an Effective Charter** ^6^	**Elements Highlighted in the AHC Charter, Informed by CFHI’s** ***Six Levers for Healthcare Improvement Framework*** ^8^
**Well-Reflected in the AHC Charter**	
Engage front-line staff early in the process to secure their buy-in and leadership	Build capacity in organizations and across all regions and provinces to research, develop, share and sustain evidence-informed and systems solutions Create and train teams to lead improvements to achieve outcomes and targets *The CFHI levers outline the importance of engaging front-line managers and providers*^8^
Built in set of quality metrics that articulate specific collaboratively identified performance indicators and extend across the continuum of care	Introduce integrated evaluation and monitoring plans, tracking progress and outcomes for team- and collaborative-level improvement Use detailed case study analysis and other learning strategies to empower impactful approaches and outcomes
Maintain CEO accountability via performance reviews linked to the organizational quality plan rather than bonus payments	Establish a network of chief executives to identify health priorities and set outcome and system improvement targets Increase the availability of timely and evidence-informed policy analysis to clarify issues and guide decisions
**Minimally Reflected in the AHC Charter**	
Engage citizens as part of the development and framing of a charter	Develop a patient- and family-centred approach to CDM *The CFHI levers outline the importance of engaging patients and citizens for improvement*^8^
Set the bar higher for citizens (including patients) and aim to improve health at a population level	No formal mention of population health in the charter, granted the focus of many initiatives went beyond disease management to prevention and promotion *The CFHI levers outline the importance of focusing on population needs*^8^
Incorporate an imperative to boost value for money, which leads to cost-savings and efficiencies	Promote sustainability of the health system Build a network of organizational, regional and provincial teams to share evidence-informed, effective, sustainable and systems-level solutions and work together to implement improvements Promote development of local channels to sustain exchange of evidence and innovation

Abbreviations: AHC, Atlantic Healthcare Collaboration for Innovation and Improvement; CFHI, Canadian Foundation for Healthcare Improvement; CDM, chronic disease management; CEO, chief executive officer.

1. Driven by persons and community
2. Create vision and build will
3. Develop capability
4. Deliver results
5. Shape culture
6. Engage across boundaries

Common elements of effective governance for collaborative healthcare improvement are evident across cited frameworks.^5-8^ Improvement efforts will only be effective if they appropriately engage those they seek to influence from the outset – frontline providers as well as patients, families and communities. Realizing improvement requires shifting accountability measures beyond the charter to a common performance improvement approach and capacity development interventions that support the change process. The AHC model included a refined governance structure that encompassed these components (Table 1), granted not all were well-reflected in the charter. Upon reflection, the QIC could have further involved patients, worked toward population health and financial returns on investment.^9^ Limited capacities in the region and a short timeframe were cited as barriers to these actions.^9^

## II. Governance Capacity

Emerson et al define collaborative governance as a series of processes and structures that engage people across boundaries of institutions to execute activities for a public purpose that could otherwise not be accomplished.^10^ The AHC was designed with a blended governance approach. While the AHCEC was primarily the accountable governing body, it also bridged connections to improvement teams. The regional teams set the foci for the chronic disease initiatives. Table 2 outlines the alignment between Ansell and Gash’s conditions for effective collaborative governance^11^ with those created by the AHCEC. Ansell and Gash argue that these factors support collective decision-making, pointing to multi-dimensional, cross-system, governance approaches, suggesting a collaborative cycle of variables that are highly iterative and nonlinear, congruent with the AHCEC.

**Table 2:** Comparison of Features of the AHCEC Processes and Activities With the Conditions and Processes of Effective Governance

**Conditions and Processes of Effective Governance** ^11^	**AHCEC’s Processes and Activities**
**Starting Conditions**	
• Power-resource-knowledge asymmetries • Incentives (and constraints) for participation • Initial and existing trust level	• Similar constraints and opportunities facing RHAs: rising rates of chronic disease prevalence, aging populations, fiscal restraints • Previous forums for CEO-level engagement and developing working relationships
**Collaborative Processes** ^a^	
• Trust-building activities • Commitment to process • Shared understanding • Intermediate outcomes (small wins) • Face-to-face dialogue	• CFHI-led regional site meetings pre-AHC to build momentum and create a platform for a pan-regional QI • Commitment to collectively identify priorities for chronic disease improvements and establish teams to implement local QI • Local-level outcomes across various phases of the collaborative, from site processes, to patient and regional-level outcomes • Quarterly meetings to discuss process and progress, including presence at four face-to-face meetings

Abbreviations: AHC, Atlantic Healthcare Collaboration for Innovation and Improvement; CFHI, Canadian Foundation for Healthcare Improvement; CDM, chronic disease management; CEO, chief executive officer; AHCEC, Atlantic Healthcare Collaboration Executive Committee; QI, quality improvement; RHA, regional health authority. ^a^ Note: institutional design (inclusiveness, transparency) and facilitative leadership (empowerment) interplay with collaborative process.

Effective governance requires a combination of multidirectional approaches.^12^ A 2011 case study of three high-performing health systems demonstrated that from a governance perspective there was strong collective and distributed senior leadership involvement,^12^ critical to fostering a QI culture.^13^ The AHCEC was integral in creating a collaborative environment for improvement across RHAs and providing top-down direction to create an environment for success.

The AHC governance approach is underlined by triangulated relationships across and between members and teams. Trust was established through working relationships, strengthened through common goal creation and priority-setting, resulting in a supportive governance approach for the QIC and its teams.

## III. Sustaining Improvement

The post-collaborative evaluation^14^ identified several facilitators for success across the designing, planning, implementing and sustaining improvement phases. Senior leadership engagement was tied to successful teams. With periods of RHA restructuring and leadership turnover, loss of momentum became a concern with the lack of senior leadership support and engagement. As the secretariat for the AHCEC, CFHI mitigated potential risks by extensively orienting newcomers to the committee.^14^ Additionally, initiatives were selected based on provincial and regional priorities, sustaining senior-level buy-in and support.

An early analysis of system priorities in the region revealed key challenges and priorities aligned across provinces, mainly those linked to chronic disease management (CDM) and patient-centred care.^15^ Notably absent were the mechanisms to connect the various organizations to work collaboratively to tackle these priorities. The AHCEC helped to formalize connections.^3^ CFHI conducted a comprehensive social network analysis to understand connections between individuals and organizations at various time-points throughout the AHC. Each survey gathered data from participants, measuring connections across three areas: who they knew, shared CDM information with, and collaborated with on CDM-related initiatives. Connections grew in all areas (spelled out in detail elsewhere),^16^ but suggesting that the AHC networks were critical drivers for success and sustainability of the CDM initiatives.^16^

Post-AHC, efforts and capacity to pursue collaborative approaches to improving healthcare were apparent. A greater number of Atlantic teams participated in CFHI-led QICs, with more than 30 organizations across seven CFHI initiatives, including four of the 11 original AHC improvement teams enrolled in subsequent CFHI QICs.^9^

Beyond continued interest in participating in QICs, some examples of continued success with local support include:

- Spread of the PEER (Peers Engaged in Education and Recovery) 126 model from Horizon Health Network in New Brunswick via the Transformational Research in Adolescent Mental Health (TRAM) network – a network established by the Canadian Institutes of Health Research (CIHR) to catalyze fundamental change in Canadian youth mental health over the next five years;
- A one-day symposium attended by all Nova Scotia Health Authority (NSHA) regions to share and spread the successes in implementing an integrated chronic disease prevention and management strategy (CDPM) across programs and services;
- Ongoing work in diabetes self-management support (SMS) across three of the four teams from Newfoundland and Labrador, eg, the team from Labrador-Grenfell Health drew on the work from Western Health to develop a diabetes registry and enhance skills and educate clinicians in diabetes SMS.^9^

The role of the Atlantic CEOs is important to continuing the QI initiated through the AHC. While CFHI continued to support teams with additional resources (eg, dissemination opportunities and continued evaluation support), this cannot replace the need for local leadership to continue to support teams and maintaining a view on common priorities.^17^

## Conclusion

The AHC demonstrates an approach to leading a pan-regional QIC with a multi-dimensional design, specifically one that encourages multiple levels of the health system to work collaboratively toward CDM. Executive-level commitment and active involvement in supporting local QIC efforts is essential; Initiatives drawn from organizational and regional strategic priorities had the support necessary from senior leadership and policy-makers to create momentum to achieve early success.

It is arguably difficult to transform public health systems to be high-performing and sustainable due to increased demands, limited resources and structural inertia.^18^ Health systems across Canada are no exception, where changes and improvement appear relatively slow and inadequately transformative.^19^ These systems may be in a state of “paradigm freeze,” unable to move past their structural limitations.^20^ Policy uncertainties and persistent dysfunctions drive systems to look for alternative strategies to achieve improvement besides reorganization or restructuring. The AHC offers an alternative approach, with the focus on building problem-solving and QI capacities within the health system, creating an enabling context for change, nurturing recognition and distributed capacities as well as a mix of bottom-up (provider- and patient-driven) dynamic with top-down (leadership) guidance and support.

## Acknowledgements

We thank the members of the Atlantic Healthcare Collaboration’s Executive Committee for their valuable contributions and support. Thanks as well to all CFHI staff involved in leading and managing the AHC, including the Medavie Health Foundation, Dartmouth, NS, Canada and Health Association Nova Scotia, Bedford, NS, Canada.

## Ethical issues

Not applicable.

## Competing interests

Authors declare that they have no competing interests.

## Authors’ contributions

All authors contributed equally to the writing of this paper.

## Authors’ affiliations

^1^Canadian Foundation for Healthcare Improvement, Ottawa, ON, Canada. ^2^Canada Research Chair on Governance and Transformation in Health Care Organizations and Systems, Université de Montréal-CRCHUM, Montreal, QC, Canada. ^3^Health Prince Edward Island, Charlottetown, PE, Canada. ^4^Canadian Patient Safety Institute, Ottawa, ON, Canada.
